# Finding Missing Heritability in Less Significant Loci and Allelic Heterogeneity: Genetic Variation in Human Height

**DOI:** 10.1371/journal.pone.0051211

**Published:** 2012-12-12

**Authors:** Ge Zhang, Rebekah Karns, Guangyun Sun, Subba Rao Indugula, Hong Cheng, Dubravka Havas-Augustin, Natalija Novokmet, Zijad Durakovic, Sasa Missoni, Ranajit Chakraborty, Pavao Rudan, Ranjan Deka

**Affiliations:** 1 Human Genetics Division, Cincinnati Children's Hospital, Cincinnati, Ohio, United States of America; 2 Center for Genome Information, Department of Environmental Health, University of Cincinnati, Cincinnati, Ohio, United States of America; 3 Institute for Anthropological Research, Zagreb, Croatia; 4 Center for Computational Genomics, Institute of Applied Genetics, University of North Texas Health Science Center, Forth Worth, Texas, United States of America; University of Texas School of Public Health, United States of America

## Abstract

Genome-wide association studies (GWAS) have identified many common variants associated with complex traits in human populations. Thus far, most reported variants have relatively small effects and explain only a small proportion of phenotypic variance, leading to the issues of ‘missing’ heritability and its explanation. Using height as an example, we examined two possible sources of missing heritability: first, variants with smaller effects whose associations with height failed to reach genome-wide significance and second, allelic heterogeneity due to the effects of multiple variants at a single locus. Using a novel analytical approach we examined allelic heterogeneity of height-associated loci selected from SNPs of different significance levels based on the summary data of the GIANT (stage 1) studies. In a sample of 1,304 individuals collected from an island population of the Adriatic coast of Croatia, we assessed the extent of height variance explained by incorporating the effects of less significant height loci and multiple effective SNPs at the same loci. Our results indicate that approximately half of the 118 loci that achieved stringent genome-wide significance (p-value<5×10^−8^) showed evidence of allelic heterogeneity. Additionally, including less significant loci (i.e., p-value<5×10^−4^) and accounting for effects of allelic heterogeneity substantially improved the variance explained in height.

## Introduction

Genome-wide association studies (GWAS) have identified more than one thousand common variants associated with complex traits in human populations [1]. However, most identified variants confer relatively small effects, and in combination explain only a small fraction of phenotypic variance [2]. Using human height as an example, a classic quantitative trait where an estimated 80∼90% of the normal variation is attributed to additive genetic factors [3]–[6], but the recently identified 180 SNPs by the GIANT (The Genetic Investigation of ANthropometric Traits) study account for only ∼10% of the overall height variance [7]. The same study also revealed two possible sources of missing heritability. First, many common variants with small effects contribute to phenotypic variation, though the strengths of association of these variants do not achieve genome-wide significance (p-value<5×10^−8^). Second, multiple variants at a single locus may jointly influence a trait (i.e. allelic heterogeneity) and explain additional phenotypic variation.

The dissection of allelic heterogeneity is complicated by the correlation between SNPs due to linkage disequilibrium (LD), which often results in multiple SNP signals in any significant locus. Usually, only the lead SNP (the SNP with smallest p-value) of a locus is reported to represent the significant association. Some GWAS have conducted conditional association analyses to identify secondary signals associated with complex traits by accounting for the effects of lead SNPs [7]–[9]. As an alternative, we propose an analytical approach that estimates allelic effects and dissects allelic heterogeneity from GWA summary data (p-values, allele frequencies and sample size) rather than requiring individual-level data.

Using this method, we have examined allelic heterogeneity in height loci that include SNPs of different significance levels based on the GIANT summary data. To assess the extent to which additional variance may be explained by incorporating multiple effect SNPs at the same loci, we studied the variance explained in a sample of 1,304 individuals collected from an island population of the Adriatic Coast of Croatia. Our results indicated that a substantial fraction of height loci showed evidence of allelic heterogeneity and by including loci of lower significance and accounting for allelic heterogeneity, we were able to explain a considerably higher proportion of trait variance.

## Methods

### Clustering significant height loci based on GIANT summary data

The height summary data from the Stage I meta-analysis of GIANT studies were downloaded from the GIANT consortium website (http://www.broadinstitute.org/collaboration/giant). The data file contains p-values, direction of effect, and number of observations at nearly 2.5 M (2,469,635) genotyped or imputed SNPs. Including SNPs with lower levels of significance may increase the variance explained [7], therefore we examined SNPs at six different levels of significance (α = 5×10^−8^ to 5×10^−3^). SNPs meeting our significance criteria were clustered into distinct loci if they were physically adjacent to one another; different lengths (500 kb∼50 kb) were used to define “physical adjacency” since lowering the significance level substantially increased the number of significant SNPs (Table 1).

**Table 1:** Numbers of significant loci and conditional signals.

Significance level	Length of context (kb)&	Number of significant loci (primary signal)	Number (%) of significant loci with secondary signal	Number (%) of significant loci with tertiary signal
5.E-08	500	118	60 (50.8%)	26 (22.0%)
5.E-07	400	151	70 (46.4%)	31 (20.5%)
5.E-06	300	217	75 (34.6%)	31 (14.3%)
5.E-05	200	354	87 (24.6%)	34 (9.6%)
5.E-04	100	781	92 (11.8%)	24 (3.1%)
5.E-03	50	2668	48 (1.8%)	10 (0.4%)

& Lowering the significance level substantially increased the number (or the density) of significant SNPs used in clustering height loci. Therefore, shorter context lengths were arbitrarily selected in defining “physical adjacency” when relaxed significance levels were used, which might artificially reduce the length of significant loci and hence the chance of allelic heterogeneity in these loci clustered at lower significance level.

### Estimation of effect sizes and allelic heterogeneous effects

The GIANT summary data do not include effect size estimates (beta coefficients), therefore we first estimated the allelic effect (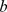) of each SNP using the following approximation function:
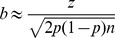
where 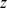 is the z-score computed from reported p-values, and is either positive or negative according to the reported direction of effect. The reference allele frequency is denoted by 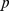 and 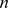 is the number of observations (sample size).

Part of the observed effect at a SNP 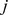 may be due to the impact of an adjacent lead SNP 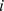 with effect (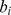). Assuming an additive effect model, this part of apparent effect of SNP 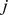 (hereafter referred to as projected effect) can be written as:
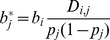
where 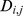 is the linkage disequilibrium coefficient between the two SNPs and 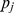 is the frequency of the reference allele SNP 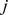. Thus, the allelic effect of SNP 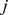 conditioned on the primary SNP 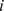 can be calculated as the difference between the observed effect and the projected effect: 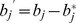 (hereafter referred to as conditional effect). Accordingly, the “conditional p-value” of SNP 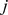 can be approximated following equation: 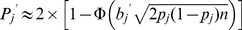
where 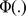 is the standard normal cumulative distribution function. For each significant locus, we selected the most significant SNP as the primary SNP and estimated the conditional effects and p-values of all adjacent SNPs using the above functions. The SNP with the smallest conditional p-value was chosen as the secondary SNP if the conditional p-value was less than a Bonferroni-corrected significance level (0.05 divided by the number of SNPs at the locus). By applying this procedure iteratively (similar to stepwise conditioning), we also obtained conditional effect estimates of tertiary and quaternary SNPs, until conditional p-values were no longer significant. Since most GIANT study samples are European in origin, we used allele frequencies and linkage disequilibrium parameters calculated from haplotype data of HapMap Phase 2 CEU samples (release 24) [10].

### Analysis of variance explained

To assess height variance explained, we used a study sample derived from a genetic study of metabolic syndrome in a relatively isolated population [11]–[14]. Briefly, study participants were recruited from Hvar, a middle Dalmatian island on the eastern Adriatic coast of Croatia. Blood samples and anthropometric data were collected in two field seasons of May 2007 and May 2008. For this study, we used the data on 1,304 individuals for whom genome-wide SNP data and height measurements were available. The study was approved by the Ethics Committee of the Institute for Anthropological Research in Zagreb, Croatia and the Institutional Review Board of the University of Cincinnati. Written informed consent was obtained from all participants.

Genome-wide SNP genotype data were obtained using the Affymetrix Human SNP Array 5.0 according to the manufacture's protocol. Genotype calls were determined using the CRLMM algorithm [15], [16]. After QC filtering (MAF>0.02, HWE p-value>0.0001, call rate>95%), we performed genotype imputation using MACH [17] and the reference haplotype data from the Phase 2 CEU HapMap, yielding a final genotype data set of 2.5 million SNPs in 1,304 individuals (565 males and 739 females).

We estimated the fraction of variance explained using genetic scores that combine information from primary SNPs and conditional SNPs selected from significant loci. The weighted genetic score was constructed as 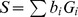; where 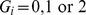 indicates the number of reference alleles for a specific SNP and 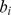 is the estimated allelic effect (for primary SNPs) or conditional effect (for secondary and tertiary SNPs) . The variance explained (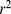) was then calculated based on a linear regression model using the constructed scores as predictor and age-, gender-, and their interaction term adjusted height residuals as outcome.

## Results

**Figure 1:**
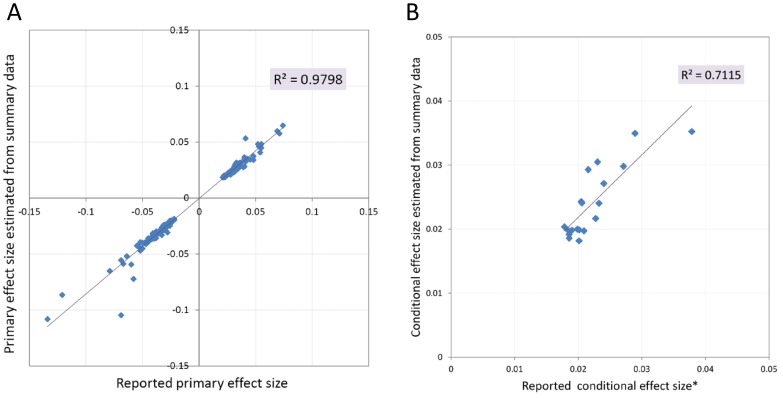
Correlation between reported and estimated effect sizes of the 180 primary height SNPs (A) and the 19 secondary SNPs (B) reported by Lango Allen et al. * The reference study did not report the effect sizes of the secondary signals. Here we used the values converted from the reported p-values based on conditional analyses in a subset of Stage 1 GIANT studies (Table 1 of Lango Allen et al).

To evaluate the validity of our proposed analytical procedure for estimating effect sizes and allelic heterogeneity from GIANT summary data, we first compared the concordance between our estimated effect sizes with the reported effect sizes listed in the GIANT meta-analysis report [7]. Of the 180 genome-wide significant SNPs, our estimated allelic effects correlated near perfectly (r^2^ = 0.98) with their reported values (Figure 1A and Table S1). Of the 19 SNPs showing significant secondary signals listed in Table 1 of [7], the conditional allelic effects estimated using our proposed procedure correlated reasonably well (r^2^ = 0.71) with their reported values (Figure 1B and Table S2). These results demonstrated that our proposed method accurately estimated effect sizes from the GIANT summary data (p-value, allele frequency and sample size). By incorporating LD information from HapMap (CEU), our procedure could also approximately dissect heterogeneous allelic effects (secondary or tertiary signals) of a significant locus.

**Figure 2:**
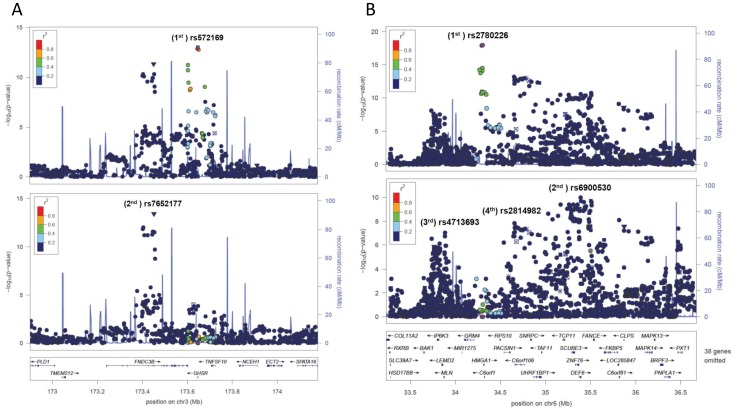
Two example loci with allelic heterogeneity. (A) The GHSR locus included a secondary signal (rs7652177) after accounting for the primary signal (rs572169). (B) The HMGA1 locus had a more complicated pattern of allelic heterogeneity; with significant secondary, tertiary and quaternary signals after multiple rounds of conditioning (only the first round of conditioning is shown). The secondary p-values (bottom plots) conditioning on the primary SNP were estimated from GIANT summary data using the analytical approach described in main text.

Using this procedure we evaluated the allelic heterogeneity of significant loci that were associated with height at different significance levels (Table 1), based on the GIANT summary data. Approximately half of the 118 significant loci that achieved stringent genome-wide significance (with p-value at the primary SNP less than 5×10^−8^) contained secondary signals (Figure 2A), and approximately one fourth had tertiary or quaternary signals after multiple rounds of conditioning (Figure 2B). All of the 18 regions with secondary signals reported by Lango Allen et al. [7] were recovered by our method (Table S3). A close examination of the significant loci with secondary signals revealed three additional features of the observed allelic heterogeneity. First, half of the identified secondary SNPs occurred within 200 kb from the primary SNPs (Figure S1). Second, we did not observe consistent LD between the primary and secondary SNPs (∼10% of secondary SNPs were in LD with primary SNPs with r^2^>0.1), and third, there was no clear correlation between the estimated effect sizes between the primary and secondary SNPs (Figure S2).

As anticipated, relaxing the significance level substantially increased the number of significant loci. However, the percentage of loci with significant secondary or tertiary signals declined as the significance of the primary SNPs decreased (Table 1). For example, when the significance level was set at 5×10^−6^, only 75 (34.6%) of the 217 significant loci included significant secondary signals, which was smaller than 50.8% – the percentage of significant loci with secondary signals identified at more stringent significance level (5×10^−8^). This decline was mainly due to the shorter sizes of the significant loci clustered by SNPs with lesser statistical significance.

**Figure 3:**
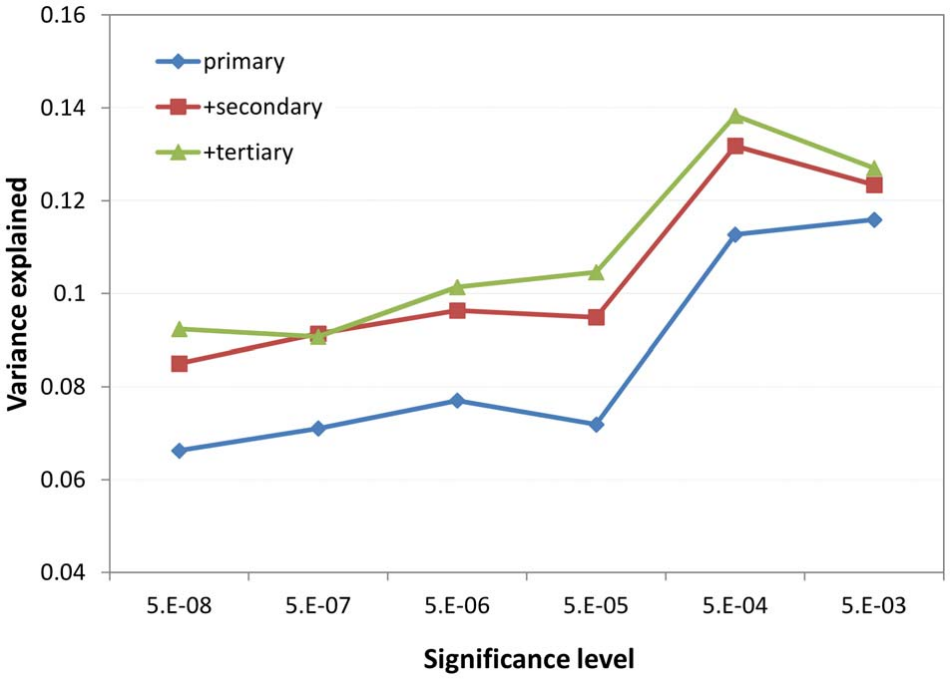
Additional fraction of variance explained could be obtained by including less significant SNPs and secondary/tertiary SNPs.

In our study samples, we assessed the extent of variance explained in age and gender adjusted height using genetic scores based on the estimated allelic effects of the clustered significant loci. Height was normally distributed in both males (N = 565) and females (N = 739) with larger variability observed in males (Figure S3). In Table 2, we listed the fractions of variance explained by various significant loci selected at different levels of significance, with or without secondary and tertiary SNPs. As demonstrated by Figure 3, two important patterns in the variance explained could be identified. First, the fraction of variance explained increased with relaxing the significance level, with the maximum around 5×10^−4^. Second, additional proportion (∼30%) of variance could be explained by including secondary and tertiary SNPs. Adding quaternary SNPs did not explain additional variance (data not shown). In our samples, the highest level of variance explained (13.8%) was achieved by the weighted-genetic score that included primary SNPs with p-value<5×10^−4^ as well as significant secondary and tertiary SNPs. The proportions of variance explain were constantly higher in females than in males (Table 2).

**Table 2:** Fraction of height variance explained.

Significance level	Number of SNPs#			All (N = 1304)			Female (N = 739)			Male (N = 565)		
	(1st)	(+2nd)	(+3rd)	(1st)	(+2nd)	(+3rd)	(1st)	(+2nd)	(+3rd)	(1st)	(+2nd)	(+3rd)
5.E-08	118	178	204	0.066	0.085	0.092	0.081	0.095	0.107	0.051	0.075	0.078
5.E-07	151	221	252	0.071	0.091	0.091	0.084	0.102	0.100	0.059	0.081	0.082
5.E-06	217	292	323	0.077	0.096	0.101	0.104	0.117	0.120	0.052	0.076	0.083
5.E-05	354	441	475	0.072	0.095	0.105	0.092	0.112	0.118	0.053	0.078	0.092
5.E-04	781	873	897	0.113	0.132	**0.138** *	0.144	0.156	**0.162** *	0.084	0.109	**0.116** *
5.E-03	2668	2716	2726	0.116	0.123	0.127	0.145	0.153	0.156	0.088	0.095	0.099

# The number of SNPs used in constructing the genetic score. (1st): primary SNPs only; (+2nd): primary+secondary SNPs; and (+3rd) primary+secondary+tertiary SNPs.

* The highest level of variance explained was achieved by including less significant SNPs plus significant secondary and tertiary SNPs.

## Discussion

GWAS have successfully established many robust associations between common variants and human complex traits, yet the extent of phenotypic variability explained by these variants remains disappointingly low. In this study, we examined two possible sources of missing heritability: first, variants associated with height at non-GWA significance, and second multiple variants at a single locus jointly influencing a trait.

Based on the summary data of the GIANT (stage 1) studies, we clustered significant SNPs selected at different significance levels into distinct significant loci and dissected the allelic effects of SNPs at each locus using a novel analytical approach that accounts for the LD between pair of SNPs. Compared to the reported results [7], our proposed method accurately estimated the effect sizes of primary SNPs and efficiently detected allelic heterogeneity in many significant loci. We observed evidence of allelic heterogeneity in roughly half of the significant loci (60 out of 118) that reached stringent genome wide significance (5×10^−8^). All of the 18 regions with second signals reported by Lango Allen et al. were recovered in our list. The larger fraction of loci with allelic heterogeneity recovered by our method (∼50% vs ∼10% by Lango Allen et al) may be due to the less stringent significance cut off for secondary signals used in the current study. We controlled multiple testing for each locus independently by a Bonferroni-corrected significance level (0.05 divided by the number of SNPs at the locus). Whereas, Lango Allen et al. used a more stringent significance level (3.3×10^−7^) for all of the 180 significant loci simultaneously.

A recent paper by Yang et al. [18] reported a conditional and joint association analysis of GWAS summary-level statistics and using the GIANT summary data identified 36 loci with multiple associated variants for height. Although targeting the same analytical problem, our method differs from their approach. Yang et al. used a multivariate approach to model the joint effects of multiple SNPs simultaneously and estimated the conditional effect iteratively over all the SNPs across the whole genome. Our method is much simpler by only considering the LD between pair of SNPs once at a time and detects secondary, tertiary SNPs in a stepwise manner within each significant locus. Second, they employed a stringent genome-wide significance level (5×10^−8^) aiming to robustly establish the significant association of the identified SNPs. While our objective was to assess the proportion of “missing heritability” that could be explained by allelic heterogeneity and therefore, we controlled multiple testing for each locus separately. Nonetheless, 33 of the 36 loci reported by Yang et al. were included in our top list of 60 loci with multiple associated SNPs (Table S3). The concordance of these results again demonstrated the validity of our analytical method.

As demonstrated by Figure 2B, the allelic heterogeneity for some significant loci might involve more than two effect variants and span several million base pairs in length, covering multiple genes. Even in the simple example (Figure 2A), the secondary SNP (rs7652177) is located in a different gene (FNDC3B) than the primary SNP (a synonymous SNP in GHSR). In addition, rs7652177 is a non-synonymous SNP and might have functional consequence. This complex pattern reflects the complexity of allelic heterogeneity in complex phenotypes, which may go beyond the traditional perception of allelic heterogeneity for Mendelian traits as “different mutations within a single gene locus cause the same disorder”, in which a “gene” is usually interpreted as a functional gene (e.g. protein-coding gene) with relatively clear structural boundaries. This complex pattern of allelic heterogeneity also suggests that the search for causal variants in a significant locus would require comprehensive examination of a broader region that extends beyond individual gene with plausible functional relevance. In addition, the primary and secondary SNPs of a single locus were likely to cluster together, usually within several hundred kb. However, there was no obvious LD or correlation of effect sizes between these SNPs, which suggested that, although physically adjacent, multiple SNPs might confer their effects to a polygenic trait in a relatively independent manner.

The 118 primary SNPs that reached genome-wide significance (p-value<5×10^−8^) explained 6.6% height variance and the 217 primary SNPs with suggestive genome-wide significance (p-value<5×10^−6^) explained 7.7% variance. These results agreed closely with the proportion of variance explained in the same study samples [19] using the 180 lead SNPs identified by the joint analysis of Stage I+II GIANT studies. Consistent with previous findings [7], including SNPs with less significant p-values increased the variance explained in age- and gender- adjusted height (Table 2 and Figure 3). The highest level of variance explained was observed when the significance level was set between (5×10^−4^ and 5×10^−3^). This observation suggests that an appreciable fraction of SNPs far from genome-wide significance might have small but genuine effects, and including these SNPs could substantially increase the variance explained. In addition, incorporating the secondary or tertiary SNPs resulted an average increase of ∼30% in height variance explained (Figure 3). This percentage is consistent with a recent report of allelic heterogeneity in *cis*-expression quantitative traits [20]. Taken together, including less significant SNPs as well as secondary and tertiary SNPs yielded the highest level of variance explained (13.8%) observed in our samples. Another interesting observation was the fractions of variance explained in female samples were consistently higher (∼50%) than in male samples (Table 2). This difference might partially be due to the larger variance in age- and gender- adjusted height in male (SD = 6.9 cm) versus the female samples (SD = 6.0 cm), or it might indicate intrinsic gender differences in effect sizes of significant SNPs.

Our study has several limitations. First, we estimated the effect sizes and allelic heterogeneity from summary data. The estimation procedure is approximate and depends largely on the assumption of additivity, including both additive allelic effects and between-SNP additive effects, although this assumption is generally supported by theoretical [21] and empirical data [7]. Second, our estimation method can not explicitly distinguish genuine allelic heterogeneity from multiple SNPs in partial LD with a functional variant – the apparently “independent” effects might be projected from a hidden functional variant. Third, because there is no consensus definition of “significant loci”, arbitrarily selected lengths were used to cluster significant SNPs, which could break down a continuous significant locus into small pieces if the length definition was short (i.e. <100 kb). Given these limitations, our analytical approach can only be regarded as a rough evaluation of the allelic effects of significant height-associated loci. Although we have not tested the accuracy of our method, its validity is supported by the increased variance explained in our independent cohort when integrating the estimated allelic heterogeneity and the close agreement between our results and those reported by Lango Allen et al [7] and Yang et al [18] (Table S3). The detailed dissection of allelic heterogeneity will require deep sequencing of the significant loci to identify the real functional variants.

In summary, we have investigated allelic heterogeneity of height-associated loci using an analytical approximation approach. Our results demonstrated that a substantial fraction of significant loci showed evidence of allelic heterogeneity and a significant proportion may involve more than two effective SNPs. We also examined the extent of height variance explained by the genetic scores constructed based on the identified significant primary and secondary/tertiary SNPs in a sample collected from an isolated eastern European population. We confirmed that including loci with lower significance levels and accounting for multiple variants at a locus considerably increased the variance explained. We anticipate that further analyses of allelic heterogeneity using sequencing technology and more accurate estimation of allelic effects through an elaborated analytical model will lead identification of additional variants with independent effects, and in turn increase the proportion of variance explained.

## Supporting Information

Figure S1 **Distribution of distance between the primary and secondary SNPs.**
(TIF)

Figure S2 **Correlation between secondary effect and primary effect.**
(TIF)

Figure S3 **Distribution of height in females (N = 739) and males (N = 565).**
(TIF)

Table S1 Effect sizes of the 180 height-associated SNPs reported by Lango Allen et al. [*Nature* 467 (7317): 832–8].
(PDF)

Table S2 Effect sizes and p-values of the 19 secondary signals reported by Lango Allen et al. [*Nature* 467 (7317): 832–8].
(PDF)

Table S3 The 60 significant loci with secondary signals (ordered by p-value of the secondary signal).
(PDF)

## References

[1] Hindorff LA, Sethupathy P, Junkins HA, Ramos EM, Mehta JP, et al (2009) Potential etiologic and functional implications of genome-wide association loci for human diseases and traits. Proc Natl Acad Sci U S A 106: 9362–9367. 19474294 10.1073/pnas.0903103106 PMC2687147

[2] Manolio TA, Collins FS, Cox NJ, Goldstein DB, Hindorff LA, et al (2009) Finding the missing heritability of complex diseases. Nature 461: 747–753. 19812666 10.1038/nature08494 PMC2831613

[3] Visscher PM (2008) Sizing up human height variation. Nat Genet 40: 489–490. 18443579 10.1038/ng0508-489

[4] Silventoinen K (2003) Determinants of variation in adult body height. Journal of Biosocial Science 35: 263–285. 12664962 10.1017/s0021932003002633

[5] Perola M, Sammalisto S, Hiekkalinna T, Martin NG, Visscher PM, et al (2007) Combined genome scans for body stature in 6,602 European twins: evidence for common Caucasian loci. PLoS Genet 3: e97. 17559308 10.1371/journal.pgen.0030097 PMC1892350

[6] Fisher RA (1918) The correlation between relatives on the supposition of Mendelian inheritance. Transactions of the Royal Society of Edinburgh 52: 399–433.

[7] Lango AH, Estrada K, Lettre G, Berndt SI, Weedon MN, et al (2010) Hundreds of variants clustered in genomic loci and biological pathways affect human height. Nature 467: 832–838. 20881960 10.1038/nature09410 PMC2955183

[8] Galarneau G, Palmer CD, Sankaran VG, Orkin SH, Hirschhorn JN, et al (2010) Fine-mapping at three loci known to affect fetal hemoglobin levels explains additional genetic variation. Nat Genet 42: 1049–1051. 21057501 10.1038/ng.707 PMC3740938

[9] Teslovich TM, Musunuru K, Smith AV, Edmondson AC, Stylianou IM, et al (2010) Biological, clinical and population relevance of 95 loci for blood lipids. Nature 466: 707–713. 20686565 10.1038/nature09270 PMC3039276

[10] Frazer KA, Ballinger DG, Cox DR, Hinds DA, Stuve LL, et al (2007) A second generation human haplotype map of over 3.1 million SNPs. Nature 449: 851–861. 17943122 10.1038/nature06258 PMC2689609

[11] Zhang G, Karns R, Narancic NS, Sun G, Cheng H, et al (2010) Common SNPs in FTO gene are associated with obesity related anthropometric traits in an island population from the eastern Adriatic coast of Croatia. PLoS One 5: e10375. 20442772 10.1371/journal.pone.0010375 PMC2860984

[12] Karns R, Zhang G, Jeran N, Havas-Augustin D, Missoni S, et al (2011) Replication of genetic variants from genome-wide association studies with metabolic traits in an island population of the Adriatic coast of Croatia. Eur J Hum Genet 19: 341–346. 21150882 10.1038/ejhg.2010.178 PMC3061997

[13] Deka R, Durakovic Z, Niu W, Zhang G, Karns R, et al (2012) Prevalence of metabolic syndrome and related metabolic traits in an island population of the Adriatic. Ann Hum Biol 39: 46–53. 22149059 10.3109/03014460.2011.637512

[14] Karns R, Zhang G, Sun G, Rao IS, Cheng H, et al (2012) Genome-wide association of serum uric Acid concentration: replication of sequence variants in an island population of the Adriatic coast of croatia. Ann Hum Genet 76: 121–127. 22229870 10.1111/j.1469-1809.2011.00698.x PMC3302578

[15] Carvalho B, Bengtsson H, Speed TP, Irizarry RA (2007) Exploration, normalization, and genotype calls of high-density oligonucleotide SNP array data. Biostatistics 8: 485–499. 17189563 10.1093/biostatistics/kxl042

[16] Carvalho BS, Louis TA, Irizarry RA (2010) Quantifying uncertainty in genotype calls. Bioinformatics 26: 242–249. 19906825 10.1093/bioinformatics/btp624 PMC2804295

[17] Li Y, Willer C, Sanna S, Abecasis G (2009) Genotype imputation. Annu Rev Genomics Hum Genet 10: 387–406. 19715440 10.1146/annurev.genom.9.081307.164242 PMC2925172

[18] Yang J, Ferreira T, Morris AP, Medland SE, Madden PA, et al (2012) Conditional and joint multiple-SNP analysis of GWAS summary statistics identifies additional variants influencing complex traits. Nat Genet 44: 369–3. 22426310 10.1038/ng.2213 PMC3593158

[19] Zhang G, Karns R, Sun G, Indugula SR, Cheng H, et al (2011) Extent of height variability explained by known height-associated genetic variants in an isolated population of the Adriatic coast of Croatia. PLoS One 6: e29475. 22216288 10.1371/journal.pone.0029475 PMC3246488

[20] Wood AR, Hernandez DG, Nalls MA, Yaghootkar H, Gibbs JR, et al (2011) Allelic heterogeneity and more detailed analyses of known loci explain additional phenotypic variation and reveal complex patterns of association. Hum Mol Genet 20: 4082–4092. 21798870 10.1093/hmg/ddr328 PMC3177649

[21] Hill WG, Goddard ME, Visscher PM (2008) Data and theory point to mainly additive genetic variance for complex traits. PLoS Genet 4: e1000008. 18454194 10.1371/journal.pgen.1000008 PMC2265475

