# Supplementary figures and images for: Finding Missing Heritability in Less Significant Loci and Allelic Heterogeneity: Genetic Variation in Human Height
Source: PLoS One. 2012 Dec 12;7(12):e51211. doi: 10.1371/journal.pone.0051211 (PMC3521016; doi:10.1371/journal.pone.0051211)

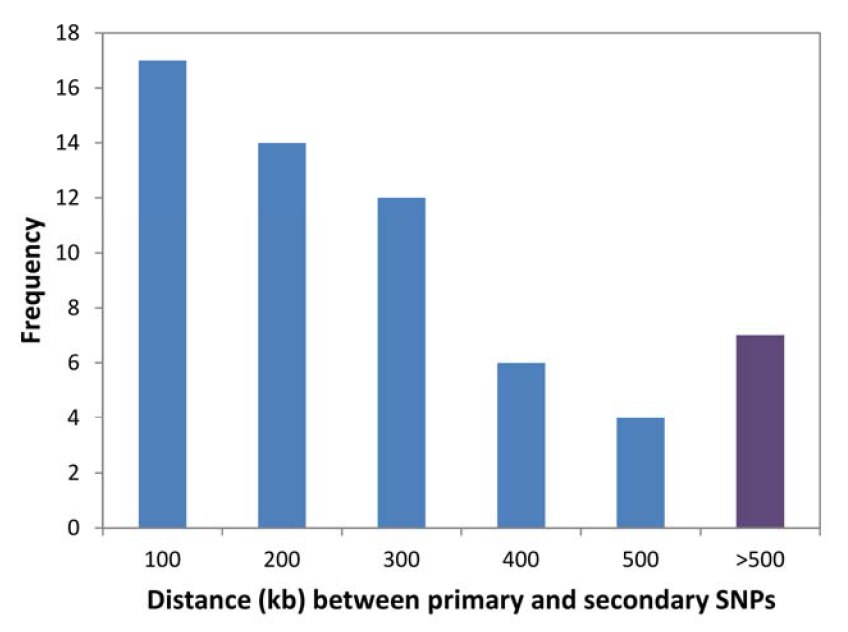

Supplement: Figure S1 — Distribution of distance between the primary and secondary SNPs. (TIF) [file pone.0051211.s001.tif]

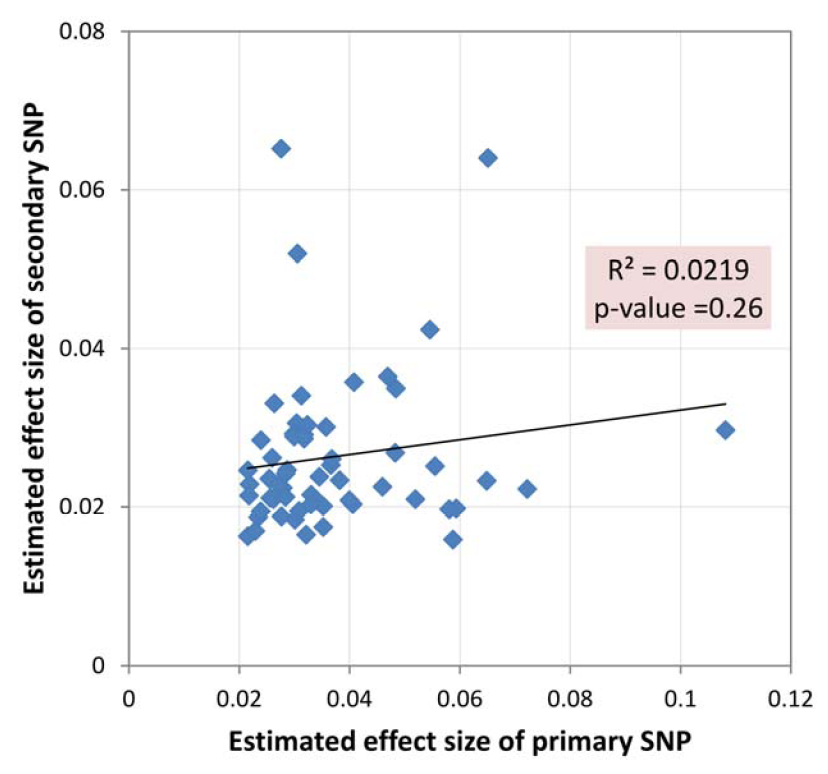

Supplement: Figure S2 — Correlation between secondary effect and primary effect. (TIF) [file pone.0051211.s002.tif]

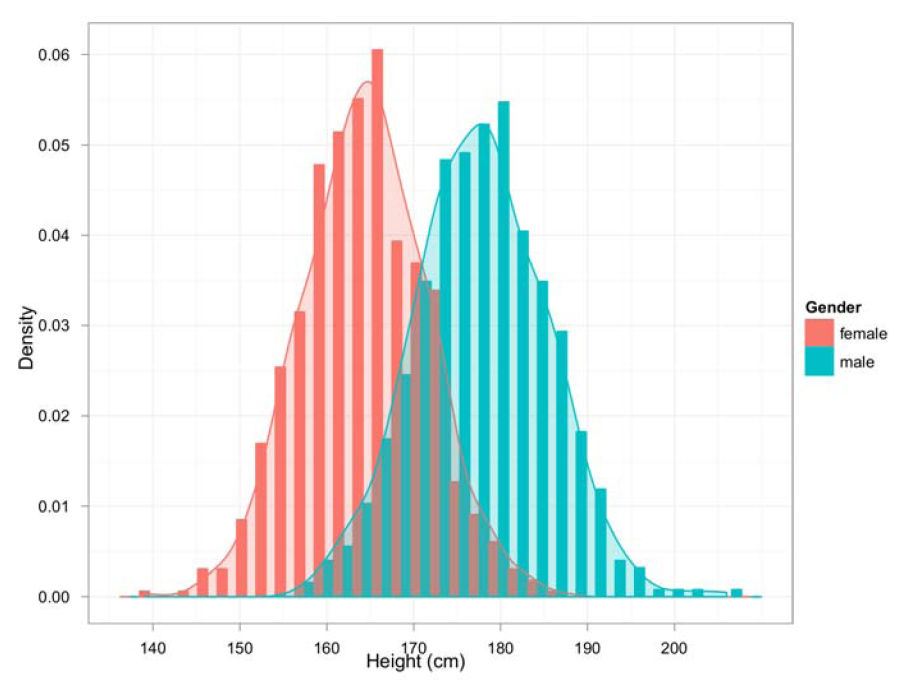

Supplement: Figure S3 — Distribution of height in females (N = 739) and males (N = 565). (TIF) [file pone.0051211.s003.tif]
