# Supplementary material for: Finding Missing Heritability in Less Significant Loci and Allelic Heterogeneity: Genetic Variation in Human Height
Source: PLoS One. 2012 Dec 12;7(12):e51211. doi: 10.1371/journal.pone.0051211 (PMC3521016; doi:10.1371/journal.pone.0051211)
Supplement: Table S1 — Effect sizes of the 180 height-associated SNPs reported by Lango Allen et al. [Nature 467 (7317): 832–8]. (PDF) [file pone.0051211.s004.pdf]

Supplementary Table S1. Effect sizes of the 180 height-associated SNPs reported by Lango Allen et al. [*Nature* 467 (7317): 832-8]

snp[@]	chr	pos	gene	alleles	freq	GIANT Stage 1[$]		Estimated[&]		
						beta	pval	freq	beta	pval
rs425277	1	2059032	PRKCZ	T/C	0.28	0.024	1.70E-06	0.28	0.021	1.65E-06
rs2284746	1	17179262	MFAP2	C/G	0.48	-0.035	5.60E-15	0.42	-0.031	5.64E-15
rs1738475	1	23409478	HTR1D	C/G	0.59	0.022	1.90E-06	0.60	0.019	1.87E-06
rs4601530	1	24916698	CLIC4	T/C	0.26	-0.024	2.00E-06	0.25	-0.021	2.02E-06
rs7532866	1	26614131	LIN28	A/G	0.67	0.022	3.30E-06	0.73	0.020	3.26E-06
rs2154319	1	41518357	SCMH1	T/C	0.75	-0.034	4.30E-10	0.78	-0.029	4.30E-10
rs17391694	1	78396214	GIPC2	T/C	0.12	0.040	5.90E-07	0.14	0.028	5.92E-07
rs6699417	1	88896031	PKN2	T/C	0.61	0.022	1.70E-06	0.63	0.019	1.68E-06
rs10874746	1	93096559	RPL5	T/C	0.37	-0.022	1.70E-06	0.34	-0.020	1.68E-06
rs9428104	1	118657110	SPAG17	A/G	0.24	-0.038	8.90E-13	0.32	-0.030	8.88E-13
rs11205277	1	148159496	SF3B4	A/G	0.58	-0.045	1.20E-18	0.62	-0.036	1.20E-18
rs17346452	1	170319910	DNM3	T/C	0.73	-0.038	3.30E-14	0.78	-0.036	3.28E-14
rs1325598	1	175058872	PAPPA2	A/G	0.43	-0.026	1.60E-08	0.49	-0.022	1.61E-08
rs1046934	1	182290152	TSEN15	A/C	0.64	-0.046	6.40E-22	0.63	-0.039	6.43E-22
rs10863936	1	210304421	DTL	A/G	0.53	-0.022	6.20E-07	0.55	-0.019	6.16E-07
rs6684205	1	216676325	TGFB2	A/G	0.71	-0.033	2.00E-11	0.81	-0.033	1.97E-11
rs11118346	1	217810342	LYPLAL1	T/C	0.47	-0.026	2.20E-09	0.45	-0.023	2.19E-09
rs10799445	1	225978506	JMJD4	A/C	0.77	0.031	1.20E-08	0.71	0.024	1.18E-08
rs4665736	2	25041103	DNAJC27	T/C	0.54	0.034	1.40E-13	0.47	0.029	1.44E-13
rs6714546	2	33214929	LTBP1	A/G	0.28	-0.025	2.20E-06	0.30	-0.020	2.21E-06
rs17511102	2	37814117	CDC42EP3	A/T	0.91	-0.060	1.30E-12	0.94	-0.059	1.26E-12
rs2341459	2	44621706	C2orf34	T/C	0.27	0.028	3.60E-08	0.28	0.024	3.58E-08
rs12474201	2	46774789	SOCS5	A/G	0.35	0.023	1.00E-06	0.32	0.020	1.04E-06
rs3791675	2	55964813	EFEMP1	T/C	0.23	-0.050	2.40E-20	0.28	-0.040	2.40E-20
rs11684404	2	88705737	EIF2AK3	T/C	0.67	-0.027	6.40E-09	0.70	-0.024	6.42E-09
rs7567288	2	134151294	NCKAP5	T/C	0.80	-0.031	6.70E-08	0.80	-0.026	6.73E-08
rs7567851	2	178392966	PDE11A	C/G	0.08	0.041	7.50E-07	0.03	0.053	7.52E-07
rs1351164	2	217980143	TNS1	T/C	0.79	0.028	3.70E-07	0.78	0.024	3.66E-07
rs12470505	2	219616613	CCDC108/IHH	T/G	0.90	0.048	1.30E-10	0.84	0.034	1.29E-10
rs2629046	2	224755988	SERPINE2	T/C	0.55	0.025	2.20E-08	0.55	0.022	2.17E-08
rs2580816	2	232506210	NPPC	T/C	0.19	-0.041	1.80E-12	0.25	-0.031	1.78E-12
rs12694997	2	241911659	2-Sep	A/G	0.24	-0.027	1.80E-07	0.26	-0.023	1.77E-07
rs2597513	3	13530836	HDAC11	T/C	0.90	-0.039	1.10E-07	0.88	-0.031	1.15E-07
rs13088462	3	51046753	DOCK3	T/C	0.94	-0.054	3.10E-07	0.94	-0.043	3.12E-07
rs2336725	3	53093779	RTF1	T/C	0.55	-0.026	3.50E-08	0.54	-0.022	3.52E-08
rs9835332	3	56642722	C3orf63	C/G	0.46	-0.022	8.70E-07	0.46	-0.019	8.74E-07

rs17806888	3	67499012	SUCLG2	T/C	0.88	0.040	1.10E-07	0.91	0.037	1.09E-07
rs9863706	3	72520103	RYBP	T/C	0.22	-0.030	1.50E-08	0.23	-0.026	1.47E-08
rs6439167	3	130533446	C3orf47	T/C	0.21	-0.034	7.20E-10	0.18	-0.031	7.20E-10
rs9844666	3	137456906	PCCB	A/G	0.25	-0.028	3.10E-08	0.14	-0.031	3.05E-08
rs724016	3	142588260	ZBTB38	A/G	0.56	-0.067	4.50E-52	0.52	-0.059	4.47E-52
rs572169	3	173648421	GHSR	T/C	0.31	0.036	9.90E-14	0.33	0.031	9.90E-14
rs720390	3	187031377	IGF2BP2	A/G	0.39	0.031	1.60E-10	0.38	0.025	1.62E-10
rs2247341	4	1671115	SLBP/FGFR3	A/G	0.36	0.025	6.80E-08	0.42	0.021	6.78E-08
rs6449353	4	17642586	LCORL	T/C	0.85	0.071	1.30E-27	0.84	0.058	1.35E-27
rs17081935	4	57518233	POLR2B	T/C	0.20	0.031	4.80E-08	0.17	0.028	4.77E-08
rs7697556	4	73734177	ADAMTS3	T/C	0.47	0.022	1.30E-06	0.51	0.019	1.35E-06
rs788867	4	82369030	PRKG2/BMP3	T/G	0.68	-0.039	1.80E-15	0.73	-0.034	1.78E-15
rs10010325	4	106325802	TET2	A/C	0.49	0.021	2.30E-06	0.43	0.018	2.33E-06
rs7689420	4	145787802	HHIP	T/C	0.16	-0.069	1.40E-29	0.19	-0.055	1.38E-29
rs955748	4	184452669	WWC2	A/G	0.24	-0.024	2.20E-06	0.31	-0.020	2.15E-06
rs1173727	5	32866278	NPR3	T/C	0.40	0.036	4.00E-15	0.48	0.030	3.97E-15
rs11958779	5	55037656	SLC38A9	A/G	0.70	-0.028	8.00E-09	0.71	-0.025	8.04E-09
rs10037512	5	88390431	MEF2C	T/C	0.56	0.027	3.80E-09	0.53	0.023	3.82E-09
rs13177718	5	108141243	FER	T/C	0.07	-0.041	4.10E-06	0.08	-0.032	4.10E-06
rs1582931	5	122685098	CEP120	A/G	0.47	-0.025	2.10E-08	0.46	-0.022	2.08E-08
rs274546	5	131727766	SLC22A5	A/G	0.40	-0.028	8.50E-10	0.34	-0.025	8.53E-10
rs526896	5	134384604	PITX1	T/G	0.73	0.032	1.90E-09	0.68	0.025	1.93E-09
rs4282339	5	168188818	SLIT3	A/G	0.20	-0.035	3.40E-10	0.18	-0.031	3.38E-10
rs12153391	5	171136043	FBXW11	A/C	0.25	-0.033	8.70E-10	0.24	-0.028	8.75E-10
rs889014	5	172916720	BOD1	T/C	0.36	-0.029	4.50E-10	0.38	-0.025	4.51E-10
rs422421	5	176449932	FGFR4/NSD1	T/C	0.22	-0.033	1.40E-09	0.22	-0.028	1.43E-09
rs6879260	5	179663620	GFPT2	T/C	0.39	-0.028	5.60E-10	0.34	-0.025	5.61E-10
rs3812163	6	7670759	BMP6	A/T	0.54	-0.037	6.70E-16	0.51	-0.031	6.66E-16
rs1047014	6	19949472	ID4	T/C	0.75	-0.029	1.10E-07	0.73	-0.023	1.13E-07
rs806794	6	26308656	Histone cluster	A/G	0.70	0.053	5.50E-26	0.73	0.046	5.54E-26
rs3129109	6	29192211	OR2J3	T/C	0.39	-0.026	3.30E-08	0.38	-0.022	3.28E-08
rs2256183	6	31488508	MICA	A/G	0.45	0.035	2.70E-14	0.56	0.030	2.67E-14
rs6457620	6	32771977	HLA locus	C/G	0.51	-0.024	3.60E-08	0.50	-0.021	3.65E-08
rs2780226	6	34307070	HMGA1	T/C	0.92	-0.079	1.00E-18	0.93	-0.065	1.02E-18
rs6457821	6	35510783	PPARD/FANCE	A/C	0.02	-0.121	1.80E-11	0.03	-0.086	1.82E-11
rs9472414	6	45054484	SUPT3H/RUNX2	A/T	0.22	-0.031	2.40E-08	0.18	-0.028	2.43E-08
rs9360921	6	76322362	SENP6	T/G	0.89	-0.048	4.60E-11	0.88	-0.040	4.56E-11
rs310405	6	81857081	FAM46A	A/G	0.52	0.030	3.60E-11	0.48	0.026	3.59E-11
rs7759938	6	105485647	LIN28B	T/C	0.68	-0.042	8.70E-18	0.64	-0.035	8.69E-18
rs1046943	6	109890634	ZBTB24	A/G	0.58	0.022	8.60E-07	0.64	0.020	8.61E-07

rs961764	6	117628849	VGLL2	C/G	0.42	-0.023	2.40E-07	0.42	-0.020	2.38E-07
rs1490384	6	126892853	C6orf173	T/C	0.50	0.037	3.20E-16	0.43	0.032	3.22E-16
rs6569648	6	130390812	L3MBTL3	T/C	0.76	-0.036	8.90E-12	0.76	-0.031	8.93E-12
rs7763064	6	142838982	GPR126	A/G	0.29	-0.045	6.40E-19	0.26	-0.039	6.41E-19
rs543650	6	152152636	ESR1	T/G	0.40	-0.032	1.40E-09	0.41	-0.025	1.36E-09
rs9456307	6	158849430	TULP4	A/T	0.06	-0.050	4.60E-07	0.05	-0.045	4.62E-07
rs798489	7	2768329	GNA12	T/C	0.30	-0.052	8.50E-25	0.28	-0.045	8.47E-25
rs4470914	7	19583047	TWISTNB	T/C	0.18	0.033	3.80E-08	0.17	0.029	3.78E-08
rs12534093	7	23469499	IGF2BP3	A/T	0.22	-0.030	5.60E-08	0.23	-0.025	5.56E-08
rs1708299	7	28156471	JAZF1	A/G	0.30	0.042	1.50E-17	0.33	0.035	1.48E-17
rs6959212	7	38094851	STARD3NL	T/C	0.32	-0.023	2.80E-06	0.29	-0.020	2.82E-06
rs42235	7	92086012	CDK6	T/C	0.31	0.055	7.30E-28	0.34	0.045	7.33E-28
rs822552	7	148281567	PDIA4	C/G	0.74	-0.030	1.30E-07	0.77	-0.024	1.32E-07
rs2110001	7	150147955	TMEM176A	C/G	0.69	-0.033	9.80E-10	0.69	-0.026	9.83E-10
rs1013209	8	24172249	ADAM28	T/C	0.25	-0.029	4.50E-08	0.21	-0.026	4.51E-08
rs7460090	8	57356717	SDR16C5	T/C	0.87	0.055	9.60E-16	0.88	0.048	9.55E-16
rs6473015	8	78341040	PEX2	A/C	0.72	-0.032	1.70E-10	0.65	-0.026	1.67E-10
rs6470764	8	130794847	GSDMC	T/C	0.20	-0.047	5.90E-17	0.19	-0.041	5.88E-17
rs12680655	8	135706519	ZFAT	C/G	0.60	0.030	4.80E-11	0.63	0.026	4.83E-11
rs7864648	9	16358732	BNC2	T/G	0.32	0.025	4.90E-07	0.34	0.021	4.87E-07
rs11144688	9	77732106	PCSK5	A/G	0.11	-0.055	1.50E-09	0.09	-0.043	1.48E-09
rs7853377	9	85742025	C9orf64	A/G	0.77	-0.026	3.10E-06	0.84	-0.025	3.06E-06
rs8181166	9	88306448	ZCCHC6	C/G	0.53	0.025	1.10E-07	0.52	0.021	1.09E-07
rs2778031	9	90025546	SPIN1	T/C	0.24	0.027	3.60E-07	0.27	0.022	3.63E-07
rs9969804	9	94468941	IPPK	A/C	0.44	0.028	5.60E-10	0.48	0.024	5.61E-10
rs1257763	9	95933766	PTPDC1	A/G	0.04	0.069	2.50E-06	0.03	0.060	2.50E-06
rs473902	9	97296056	PTCH1/FANCC	T/G	0.92	0.074	1.70E-14	0.94	0.065	1.70E-14
rs7027110	9	108638867	ZNF462	A/G	0.23	0.034	1.30E-10	0.26	0.028	1.34E-10
rs1468758	9	112846903	LPAR1	T/C	0.25	-0.026	1.50E-06	0.30	-0.020	1.53E-06
rs751543	9	118162163	PAPPA	T/C	0.72	0.029	4.50E-08	0.69	0.023	4.51E-08
rs7466269	9	132453905	FUBP3	A/G	0.64	0.036	1.20E-14	0.67	0.032	1.17E-14
rs7849585	9	138251691	QSOX2	T/G	0.33	0.032	3.40E-11	0.32	0.028	3.43E-11
rs7909670	10	12958770	CCDC3	T/C	0.44	-0.022	1.30E-06	0.51	-0.019	1.35E-06
rs2145998	10	80791702	PPIF	A/T	0.49	-0.025	2.70E-08	0.47	-0.022	2.68E-08
rs11599750	10	101795432	CPN1	T/C	0.38	-0.023	7.60E-07	0.42	-0.020	7.60E-07
rs2237886	11	2767307	KCNQ1	T/C	0.11	0.043	3.10E-08	0.11	0.035	3.12E-08
rs7926971	11	12654616	TEAD1	A/G	0.55	-0.024	7.30E-08	0.55	-0.021	7.27E-08
rs1330	11	17272605	NUCB2	T/C	0.35	0.024	4.40E-07	0.39	0.020	4.37E-07
rs10838801	11	48054856	PTPRJ/SLC39A13	A/G	0.69	-0.031	1.80E-10	0.68	-0.026	1.76E-10
rs1814175	11	49515748	FOLH1	T/C	0.34	0.023	2.60E-06	0.39	0.019	2.55E-06

rs5017948	11	51270794	OR4A5	A/T	0.18	0.027	4.70E-06	0.23	0.021	4.68E-06
rs3782089	11	65093395	SSSCA1	T/C	0.06	-0.058	5.90E-09	0.03	-0.072	5.89E-09
rs7112925	11	66582736	RHOD	T/C	0.35	-0.023	8.50E-07	0.36	-0.020	8.49E-07
rs634552	11	74959700	SERPINH1	T/G	0.14	0.041	1.40E-09	0.16	0.033	1.35E-09
rs494459	11	118079885	TREH	T/C	0.41	0.021	4.90E-06	0.34	0.019	4.93E-06
rs654723	11	128091365	FLI1	A/C	0.62	0.024	6.70E-07	0.63	0.020	6.75E-07
rs2856321	12	11747040	ETV6	A/G	0.64	-0.030	1.50E-10	0.61	-0.025	1.48E-10
rs10770705	12	20748734	SLCO1C1	A/C	0.33	0.031	4.60E-11	0.35	0.027	4.62E-11
rs2638953	12	28425682	CCDC91	C/G	0.68	0.036	8.40E-14	0.63	0.030	8.45E-14
rs2066807	12	55026949	STAT2	C/G	0.93	-0.052	9.60E-09	0.91	-0.039	9.61E-09
rs1351394	12	64638093	HMGA2	T/C	0.49	0.054	7.80E-34	0.48	0.047	7.78E-34
rs10748128	12	68113925	FRS2	T/G	0.35	0.035	3.80E-11	0.36	0.027	3.76E-11
rs11107116	12	92502635	SOCS2	T/G	0.22	0.052	1.70E-23	0.20	0.048	1.75E-23
rs7971536	12	100897919	CCDC53/GNPTAB	A/T	0.46	-0.025	1.10E-07	0.53	-0.021	1.09E-07
rs11830103	12	122389499	SBNO1	A/G	0.78	-0.035	3.80E-10	0.80	-0.030	3.79E-10
rs7332115	13	32045548	PDS5B/BRCA2	T/G	0.62	-0.025	7.60E-08	0.66	-0.022	7.64E-08
rs3118905	13	50003335	DLEU7	A/G	0.29	-0.052	3.00E-25	0.24	-0.047	2.99E-25
rs7319045	13	90822575	GPC5	A/G	0.40	0.029	4.50E-10	0.40	0.025	4.51E-10
rs1950500	14	23900690	NFATC4	T/C	0.29	0.032	3.90E-11	0.23	0.030	3.94E-11
rs2093210	14	60027032	SIX6	T/C	0.58	-0.034	2.30E-12	0.61	-0.028	2.30E-12
rs1570106	14	67882868	RAD51L1	T/C	0.20	-0.026	4.90E-06	0.21	-0.022	4.94E-06
rs862034	14	74060499	LTBP2	A/G	0.36	-0.023	1.10E-06	0.43	-0.019	1.06E-06
rs7155279	14	91555634	TRIP11	T/G	0.36	-0.029	8.90E-10	0.34	-0.025	8.91E-10
rs16964211	15	49317787	CYP19A1	A/G	0.05	-0.051	2.50E-06	0.04	-0.046	2.49E-06
rs7178424	15	60167551	C2CD4A	T/C	0.47	-0.024	2.20E-07	0.48	-0.020	2.15E-07
rs10152591	15	67835211	TLE3	A/C	0.91	0.045	3.50E-08	0.89	0.034	3.54E-08
rs12902421	15	69948457	MYO9A	T/C	0.97	-0.069	1.70E-06	0.99	-0.105	1.68E-06
rs5742915	15	72123686	PML	T/C	0.54	-0.031	3.00E-10	0.45	-0.025	2.99E-10
rs11259936	15	82371586	ADAMTSL3	A/C	0.48	-0.042	2.20E-21	0.53	-0.037	2.18E-21
rs16942341	15	87189909	ACAN	T/C	0.03	-0.134	1.30E-17	0.03	-0.108	1.28E-17
rs2871865	15	97012419	IGF1R	C/G	0.88	0.054	1.10E-12	0.87	0.041	1.07E-12
rs4965598	15	98577137	ADAMTS17	T/C	0.68	-0.035	1.40E-13	0.72	-0.032	1.36E-13
rs11648796	16	732191	NARFL	A/G	0.74	-0.031	2.40E-07	0.73	-0.024	2.40E-07
rs26868	16	2189377	CASKIN1	A/T	0.46	0.030	3.50E-08	0.43	0.023	3.47E-08
rs1659127	16	14295806	MKL2	A/G	0.34	0.024	2.90E-06	0.30	0.020	2.86E-06
rs8052560	16	87304743	CTU2/GALNS	A/C	0.79	0.039	1.40E-08	0.78	0.028	1.45E-08
rs4640244	17	21224816	KCNJ12	A/G	0.61	0.028	2.00E-07	0.63	0.021	2.00E-07
rs3110496	17	24941897	ANKRD13B	A/G	0.33	-0.023	1.60E-06	0.32	-0.020	1.58E-06
rs3764419	17	26188149	ATAD5/RNF135	A/C	0.39	-0.037	8.90E-16	0.38	-0.032	8.87E-16
rs17780086	17	27367395	LRRC37B	A/G	0.15	0.035	4.40E-08	0.17	0.028	4.39E-08

rs1043515	17	34175722	PIP4K2B	A/G	0.45	-0.022	1.30E-06	0.43	-0.019	1.35E-06
rs4986172	17	40571807	ACBD4	T/C	0.35	-0.028	7.10E-09	0.33	-0.024	7.12E-09
rs2072153	17	44745013	ZNF652	C/G	0.30	0.026	6.70E-08	0.30	0.023	6.68E-08
rs4605213	17	46599746	NME2	C/G	0.34	0.023	9.30E-07	0.40	0.019	9.32E-07
rs227724	17	52133816	NOG	A/T	0.65	-0.027	1.20E-08	0.69	-0.024	1.18E-08
rs2079795	17	56851431	TBX2	T/C	0.33	0.040	1.20E-16	0.30	0.035	1.22E-16
rs2665838	17	59320197	CSH1/GH1	C/G	0.73	-0.037	2.00E-13	0.68	-0.031	2.02E-13
rs11867479	17	65601802	KCNJ16/KCNJ2	T/C	0.34	0.024	4.90E-07	0.33	0.021	4.87E-07
rs4800452	18	18981609	CABLES1	T/C	0.79	0.048	2.40E-17	0.74	0.037	2.36E-17
rs9967417	18	45213498	DYM	C/G	0.58	-0.038	2.60E-16	0.58	-0.032	2.57E-16
rs17782313	18	56002077	MC4R	T/C	0.76	-0.025	3.50E-06	0.72	-0.020	3.48E-06
rs12982744	19	2128193	DOT1L	C/G	0.60	-0.033	2.80E-12	0.63	-0.028	2.79E-12
rs7507204	19	3379834	NFIC	C/G	0.24	0.028	2.30E-07	0.22	0.024	2.25E-07
rs891088	19	7135762	INSR	A/G	0.74	-0.025	1.70E-06	0.73	-0.021	1.72E-06
rs4072910	19	8550031	ADAMTS10	C/G	0.46	-0.029	2.50E-07	0.38	-0.025	2.52E-07
rs2279008	19	17144303	MYO9B	T/C	0.74	0.031	2.40E-07	0.74	0.023	2.40E-07
rs17318596	19	46628935	ATP5SL	A/G	0.36	0.029	3.00E-09	0.37	0.024	3.01E-09
rs1741344	20	4049800	SMOX	T/C	0.63	-0.026	3.50E-08	0.61	-0.022	3.52E-08
rs2145272	20	6574218	BMP2	A/G	0.65	-0.039	5.90E-16	0.64	-0.033	5.87E-16
rs7274811	20	31796842	ZNF341	T/G	0.23	-0.040	6.80E-14	0.20	-0.036	6.83E-14
rs143384	20	33489170	GDF5	A/G	0.58	-0.064	4.90E-39	0.60	-0.052	4.94E-39
rs237743	20	47336426	ZNFX1	A/G	0.21	0.034	7.20E-10	0.31	0.026	7.20E-10
rs2834442	21	34612656	KCNE2	A/T	0.65	0.027	7.30E-09	0.63	0.023	7.30E-09
rs4821083	22	31386341	SYN3	T/C	0.84	0.033	4.80E-08	0.88	0.032	4.79E-08

[@] The 180 height-associated SNPs and their chromosome positions and gene annotations were obtained from Supplementary Table 1 of Lango Allen et al. [*Nature* 467 (7317): 832-8]

[$] The effect sizes and p-values of the reference study were extracted from the column [STAGE 1] of Supplementary Table 1 of Lango Allelen et al.

[&] The effect sizes were estimated from the summary result data (p-values) from Stage I meta-analysis of GIANT studies using the analytical approach described in main text.
