# Supplementary material for: Finding Missing Heritability in Less Significant Loci and Allelic Heterogeneity: Genetic Variation in Human Height
Source: PLoS One. 2012 Dec 12;7(12):e51211. doi: 10.1371/journal.pone.0051211 (PMC3521016; doi:10.1371/journal.pone.0051211)
Supplement: Table S2 — Effect sizes and p-values of the 19 secondary signals reported by Lango Allen et al. [Nature 467 (7317): 832–8]. (PDF) [file pone.0051211.s005.pdf]

Supplementary Table S2. Effect sizes and p-values of the 19 secondary signals reported by Lango Allen et al. [*Nature* 467 (7317): 832-8]

Secondary SNP[@]	Primary SNP	chr	pos	gene	dist	r2	GIANT (Stage 1)[$]			Estimated[&]		
							pval	z-score	beta	pval	z-score	beta
rs2280470	rs16942341	15	87196630	ACAN	6721	0.009	1.00E-14	7.739	0.027	3.57E-13	7.271	0.030
rs10859563	rs11107116	12	92644470	SOCS2	141835	0.003	3.00E-12	6.978	0.023	7.03E-10	6.165	0.024
rs750460	rs5742915	15	72028559	PML	95127	0.004	4.00E-12	6.937	0.024	2.16E-11	6.695	0.027
rs6938239	rs2780226	6	34791613	HMGA1	484583	0.019	6.00E-12	6.880	0.038	4.44E-08	5.472	0.035
rs7652177	rs572169	3	173451771	GHSR	196650	0.006	7.00E-11	6.521	0.022	3.77E-14	7.569	0.029
rs7916441	rs2145998	10	80595583	PPIF	196119	0.112	6.00E-10	6.190	0.020	3.33E-10	6.283	0.024
rs3792752	rs1173727	5	32804391	NPR3	61887	0.020	7.00E-10	6.166	0.023	3.01E-12	6.977	0.030
rs10958476	rs7460090	8	57258362	SDR16C5	98355	0.020	1.00E-09	6.109	0.029	4.52E-10	6.235	0.035
rs2353398	rs7689420	4	145742208	HHIP	45594	0.022	2.00E-09	5.998	0.020	2.44E-07	5.162	0.020
rs2724475	rs6449353	4	17555530	LCORL	87056	0.098	2.00E-09	5.998	0.021	1.27E-06	4.844	0.020
rs2070776	rs2665838	17	59361230	GH region	41033	0.150	9.00E-09	5.749	0.020	1.46E-06	4.817	0.020
rs1401796	rs227724	17	52194758	NOG	60942	0.005	2.00E-08	5.612	0.019	7.19E-07	4.956	0.019
rs4711336	rs2780226	6	33767024	HMGA1	540046	0.111	3.00E-08	5.541	0.019	2.14E-06	4.740	0.019
rs6892884	rs12153391	5	170948228	FBXW11	187815	0.000	4.00E-08	5.491	0.020	2.55E-05	4.210	0.018
rs1367226	rs3791675	2	55943044	EFEMP1	21769	0.204	4.00E-08	5.491	0.019	9.74E-07	4.897	0.020
rs2421992	rs17346452	1	170507874	DNM3	187964	0.019	5.00E-08	5.451	0.021	6.42E-08	5.407	0.024
rs225694	rs7763064	6	142568835	GPR126	270147	0.001	1.00E-07	5.327	0.023	1.43E-05	4.339	0.022
rs10187066	rs12470505	2	219223003	IHH	393610	0.022	2.00E-07	5.199	0.018	1.49E-06	4.813	0.020
rs879882	rs2256183	6	31247431	MICA	241077	0.016	2.00E-07	5.199	0.018	3.87E-07	5.075	0.020

[@] The 19 SNPs shown secondary signals and their information were obtained from Table 1 of Lango Allen et al. [*Nature* 467 (7317): 832-8]

[$] The z-scores and effect sizes of the secondary signals were converted from the reported p-values (after conditioning), which was calculated based on conditional analyses in a subset of Stage 1 GIANT studies.

[&] The “conditional” p-values, z-scores and the effect sizes were estimated from the summary data (p-values) from Stage I meta-analysis of GIANT studies.
