# Supplementary material for: Finding Missing Heritability in Less Significant Loci and Allelic Heterogeneity: Genetic Variation in Human Height
Source: PLoS One. 2012 Dec 12;7(12):e51211. doi: 10.1371/journal.pone.0051211 (PMC3521016; doi:10.1371/journal.pone.0051211)
Supplement: Table S3 — The 60 significant loci with secondary signals (ordered by p-value of the secondary signal). (PDF) [file pone.0051211.s006.pdf]

Supplementary Table S3. The 60 significant loci with secondary signals (ordered by p-value of the secondary signal)

Locus position[@]				Primary SNP[&]						Secondary SNP[&]							Between-SNP	
chr	start	end	length	snp1	pos	freq	beta	z-score	pval	snp2	pos	freq	beta	z-score	pval	pval-un*	dist	r2
3	***172901908***	***174181163***	***1279256***	***rs572169***	***173648421***	***0.33***	***0.031***	***7.442***	***9.90E-14***	***rs7652177***	***173451771***	***0.53***	***0.029***	***7.569***	***3.77E-14***	***4.77E-12***	***196650***	***0.008***
15	***86683538***	***87716251***	***1032714***	***rs16942341***	***87189909***	***0.98***	***0.108***	***8.546***	***1.28E-17***	***rs2293087***	***87186155***	***0.43***	***0.030***	***7.545***	***4.51E-14***	***3.03E-09***	***3754***	***0.035***
15	97830681	99122005	1291325	rs4965598	98577137	0.28	0.032	7.400	1.36E-13	rs12595247	98359212	0.58	0.029	7.451	9.28E-14	3.34E-09	217925	0.043
5	***32225475***	***33897892***	***1672418***	***rs1173727***	***32866278***	***0.48***	***0.030***	***7.856***	***3.97E-15***	***rs1173735***	***32807136***	***0.27***	***0.031***	***6.997***	***2.61E-12***	***2.38E-09***	***59142***	***0.017***
12	***91985241***	***93225695***	***1240455***	***rs11107116***	***92502635***	***0.20***	***0.048***	***9.986***	***1.75E-23***	***rs11107193***	***92720025***	***0.58***	***0.027***	***6.848***	***7.49E-12***	2.47E-07	***217390***	***0.029***
2	232004246	233659407	1655162	rs2580816	232506210	0.75	0.031	7.051	1.78E-12	rs7571716	233149664	0.24	0.030	6.639	3.16E-11	8.04E-09	643454	0.015
6	7125527	8174613	1049087	rs3812163	7670759	0.49	0.031	8.077	6.66E-16	rs4371882	7738168	0.17	0.034	6.564	5.25E-11	1.46E-06	67409	0.047
6	80499662	82464278	1964617	rs310402	81857211	0.48	0.026	6.708	1.97E-11	rs806851	81087550	0.36	0.026	6.501	8.00E-11	1.93E-08	769661	0.017
6	***33236841***	***36676019***	***3439179***	***rs2780226***	***34307070***	***0.08***	***0.065***	***8.833***	***1.02E-18***	***rs6900530***	***35388949***	***0.96***	***0.064***	***6.468***	***9.92E-11***	***2.01E-08***	***1081879***	***0.010***
10	***80284066***	***81302857***	***1018792***	***rs7332***	***80784066***	***0.53***	***0.022***	***5.561***	***2.68E-08***	***rs779933***	***80588523***	***0.52***	***0.025***	***6.367***	***1.93E-10***	***4.34E-07***	***195543***	***0.056***
4	***144981449***	***146894371***	***1912923***	***rs7689420***	***145787802***	***0.81***	***0.055***	***11.296***	***1.38E-29***	***rs6537302***	***145807566***	***0.39***	***0.025***	***6.350***	***2.16E-10***	***1.54E-20***	***19764***	***0.068***
13	48929768	50641907	1712140	rs1327646	50020366	0.83	0.055	10.447	1.51E-25	rs4942875	49308504	0.09	0.042	6.286	3.25E-10	1.97E-04	711862	0.060
8	***56609100***	***57873236***	***1264137***	***rs7460090***	***57356717***	***0.88***	***0.048***	***8.032***	***9.55E-16***	***rs10958476***	***57258362***	***0.14***	***0.035***	***6.235***	***4.52E-10***	***1.29E-13***	***98355***	***0.022***
**6**	**30604111**	**32759200**	**2155090**	**rs2256183**	**31488508**	**0.56**	**0.030**	**7.613**	**2.67E-14**	**rs9262638**	**31133968**	**0.78**	**0.029**	**6.230**	**4.65E-10**	**1.21E-09**	**354540**	**0.000**
15	81555408	83249322	1693915	rs11259936	82371586	0.48	0.037	9.496	2.18E-21	rs2277849	82430354	0.72	0.026	6.057	1.39E-09	1.00E-09	58768	0.000
2	24061900	25836474	1774575	rs4665736	25041103	0.47	0.029	7.392	1.44E-13	rs11694842	25336474	0.66	0.025	6.054	1.42E-09	5.61E-09	295371	0.001
17	***58499732***	***59862169***	***1362438***	***rs2727300***	***59319130***	***0.32***	***0.031***	***7.347***	***2.02E-13***	***rs2137143***	***59159133***	***0.05***	***0.052***	***5.759***	***8.48E-09***	***3.64E-06***	***159997***	***0.024***
12	27655970	29130894	1474925	rs2638953	28425682	0.63	0.030	7.463	8.45E-14	rs11049294	28072670	0.18	0.029	5.744	9.23E-09	5.66E-04	353012	0.096
1	***169798655***	***171048063***	***1249409***	***rs17346452***	***170319910***	***0.22***	***0.036***	***7.587***	***3.28E-14***	***rs12411264***	***170460291***	***0.16***	***0.030***	***5.684***	***1.32E-08***	***1.39E-09***	***140381***	***0.002***
9	96749415	97920043	1170629	rs473902	97296056	0.94	0.065	7.671	1.70E-14	rs10512248	97299524	0.32	0.023	5.574	2.49E-08	5.31E-12	3468	0.029
9	108097068	109185808	1088741	rs7027110	108638867	0.26	0.028	6.423	1.34E-10	rs902143	108221732	0.48	0.021	5.500	3.80E-08	1.86E-05	417135	0.036
12	64070603	65177663	1107061	rs1351394	64638093	0.48	0.047	12.125	7.78E-34	rs7972091	64414914	0.09	0.036	5.446	5.16E-08	1.80E-03	223179	0.037
20	31875752	34793356	2917605	rs143384	33489170	0.40	0.052	13.069	4.94E-39	rs6058202	33241644	0.55	0.021	5.403	6.56E-08	2.34E-15	247526	0.037
5	***170307702***	***171767898***	***1460197***	***rs12153391***	***171136043***	***0.76***	***0.028***	***6.131***	***8.75E-10***	***rs4868126***	***171216074***	***0.65***	***0.022***	***5.390***	***7.05E-08***	***1.13E-09***	***80031***	***0.014***
1	216163084	217201410	1038327	rs6684205	216676325	0.19	0.033	6.708	1.97E-11	rs11118171	217114492	0.65	0.022	5.317	1.05E-07	9.70E-08	438167	0.000
2	***218591365***	***220227272***	***1635908***	***rs6741325***	***219615943***	***0.84***	***0.034***	***6.429***	***1.29E-10***	***rs1541777***	***219295535***	***0.62***	***0.021***	***5.238***	***1.62E-07***	***8.58E-09***	***320408***	***0.006***
11	46853230	48566524	1713295	rs10838801	48054856	0.33	0.026	6.381	1.76E-10	rs7102372	47453403	0.10	0.033	5.131	2.89E-07	5.47E-05	601453	0.030
2	37314117	38345500	1031384	rs17511102	37814117	0.06	0.059	7.098	1.26E-12	rs6544089	37612249	0.46	0.020	5.111	3.20E-07	6.96E-05	201868	0.025
15	96512419	97537428	1025010	rs2871865	97012419	0.87	0.041	7.121	1.07E-12	rs8039419	97339780	0.08	0.036	5.104	3.32E-07	3.23E-02	327361	0.171
2	***55340572***	***56552566***	***1211995***	***rs3791679***	***55950396***	***0.73***	***0.041***	***9.336***	***1.00E-20***	***rs934278***	***55923856***	***0.62***	***0.020***	***5.102***	***3.36E-07***	***3.54E-01***	***26540***	***0.200***
17	56333558	57353032	1019475	rs9905385	56853032	0.30	0.035	8.344	7.21E-17	rs11654397	56988387	0.42	0.020	5.088	3.62E-07	3.02E-05	135355	0.012

15	***71502604***	***72623686***	***1121083***	***rs4886782***	***72015863***	***0.73***	***0.027***	***6.156***	***7.45E-10***	***rs4886868***	***72140614***	***0.75***	***0.023***	***5.031***	***4.87E-07***	***4.87E-07***	***124751***	***0.000***
17	***51600338***	***52633903***	***1033566***	***rs227724***	***52133816***	***0.31***	***0.024***	***5.702***	***1.18E-08***	***rs4794665***	***52205328***	***0.53***	***0.019***	***5.024***	***5.06E-07***	***8.86E-08***	***71512***	***0.003***
9	137751691	138943132	1191442	rs7849585	138251691	0.32	0.028	6.627	3.43E-11	rs4266763	138409646	0.53	0.019	4.861	1.17E-06	5.16E-06	157955	0.002
18	44312287	45774588	1462302	rs9967417	45213498	0.42	0.032	8.192	2.57E-16	rs4939800	44520172	0.89	0.030	4.855	1.21E-06	1.13E-03	693326	0.038
4	***16890307***	***18160121***	***1269815***	***rs2061455***	***17644348***	***0.84***	***0.058***	***10.962***	***5.81E-28***	***rs2011603***	***17634582***	***0.34***	***0.020***	***4.841***	***1.29E-06***	***1.39E-16***	***9766***	***0.098***
20	5859203	7085234	1226032	rs6140050	6580901	0.36	0.033	8.176	2.94E-16	rs13037957	6597874	0.30	0.020	4.789	1.67E-06	6.42E-06	16973	0.001
6	75721309	76985215	1263907	rs9350591	76298247	0.14	0.037	6.593	4.32E-11	rs3822957	76664000	0.84	0.025	4.773	1.81E-06	2.57E-05	365753	0.007
1	147642748	148673037	1030290	rs7534365	148142748	0.17	0.032	5.930	3.03E-09	rs4926400	148198165	0.88	0.029	4.753	2.00E-06	1.03E-07	55417	0.009
3	186527003	187533011	1006009	rs720390	187031377	0.38	0.025	6.394	1.62E-10	rs6784185	186955759	0.82	0.024	4.718	2.38E-06	1.50E-01	75618	0.263
6	***142183299***	***143408538***	***1225240***	***rs262115***	***142859100***	***0.71***	***0.038***	***8.979***	***2.74E-19***	***rs225744***	***142604110***	***0.82***	***0.023***	***4.681***	***2.85E-06***	***4.53E-05***	***254990***	***0.005***
6	151652636	152898312	1245677	rs4870056	152203920	0.40	0.023	5.934	2.95E-09	rs3020418	152386855	0.34	0.019	4.596	4.31E-06	7.12E-09	182935	0.040
5	122185098	123285617	1100520	rs1582931	122685098	0.54	0.022	5.605	2.08E-08	rs337117	122623490	0.22	0.021	4.540	5.62E-06	1.21E-04	61608	0.016
5	133859721	134888919	1029199	rs537930	134376602	0.71	0.026	6.026	1.68E-09	rs10037018	134545835	0.22	0.021	4.505	6.63E-06	1.15E-04	169233	0.012
1	174558872	175569389	1010518	rs1325598	175058872	0.51	0.022	5.649	1.61E-08	rs2861745	174599256	0.83	0.023	4.497	6.90E-06	1.00E-05	459616	0.000
2	44121706	45123466	1001761	rs2341459	44621706	0.28	0.024	5.511	3.58E-08	rs17032525	44760835	0.10	0.028	4.415	1.01E-05	1.04E-03	139129	0.042
6	129887528	130945208	1057681	rs6569648	130390812	0.24	0.031	6.823	8.93E-12	rs1415701	130387528	0.75	0.019	4.368	1.26E-05	4.36E-11	3284	0.106
1	118151285	119209896	1058612	rs17038182	118669928	0.68	0.030	7.235	4.66E-13	rs1359551	118376700	0.70	0.018	4.340	1.42E-05	4.77E-03	293228	0.045
9	117662163	118662163	1000001	rs751543	118162163	0.69	0.023	5.470	4.51E-08	rs2416564	118410500	0.41	0.017	4.325	1.52E-05	1.38E-05	248337	0.000
11	2258666	3267307	1008642	rs234886	2758666	0.11	0.035	5.586	2.32E-08	rs10831695	2259872	0.35	0.018	4.321	1.56E-05	1.30E-04	498794	0.008
5	175897344	176956168	1058825	rs422421	176449932	0.78	0.028	6.052	1.43E-09	rs10516138	176294920	0.85	0.024	4.315	1.60E-05	5.46E-07	155012	0.014
3	52593779	53593779	1000001	rs2336725	53093779	0.46	0.022	5.513	3.52E-08	rs2581824	52997448	0.46	0.016	4.216	2.48E-05	5.22E-02	96331	0.168
12	54416334	55540089	1123756	rs703830	54988139	0.09	0.040	5.792	6.97E-09	rs2657896	55215961	0.81	0.021	4.191	2.78E-05	9.96E-02	227822	0.187
12	122867179	123867179	1000001	rs1809889	123367179	0.24	0.028	6.003	1.93E-09	rs12809142	123226597	0.02	0.065	4.175	2.98E-05	1.83E-04	140582	0.005
6	104971114	106069569	1098456	rs314263	105499438	0.37	0.034	8.595	8.31E-18	rs9377686	105613604	0.85	0.024	4.168	3.08E-05	3.93E-01	114166	0.165
17	25552984	26784025	1231042	rs3764419	26188149	0.63	0.032	8.042	8.87E-16	rs2066713	25575791	0.62	0.017	4.162	3.15E-05	1.72E-02	612358	0.049
6	25712427	26946676	1234250	rs806794	26308656	0.73	0.046	10.542	5.54E-26	rs13194984	26608542	0.16	0.023	4.147	3.37E-05	1.64E-06	299886	0.004
7	149639653	150799447	1159795	rs2110001	150147955	0.31	0.026	6.112	9.83E-10	rs743507	150338421	0.82	0.021	4.084	4.43E-05	4.45E-06	190466	0.007
3	142019720	143321331	1301612	rs724016	142588260	0.48	0.059	15.185	4.47E-52	rs2640006	142829743	0.56	0.016	4.065	4.81E-05	6.51E-02	241483	0.022
11	64593395	65593395	1000001	rs3782089	65093395	0.98	0.072	5.820	5.89E-09	rs239256	64743967	0.87	0.022	3.904	9.47E-05	8.17E-08	349428	0.063

[@] The loci were clustered from SNPs that achieved genome-wide significance (p-value $< 5\times10^{-8}$) based on Stage I meta-analysis of GIANT studies.

[&] The effect sizes of the primary and secondary SNPs were estimated from the summary data (p-values) from Stage I meta-analysis of GIANT studies.

*pval-un is the unconditional p-value extracted from the summary data (p-values) from Stage I meta-analysis of GIANT studies.

The rows highlighted in bold and italic were the 18 regions with secondary signals reported by Lango Allen et al.

The underlined rows were the 33 regions with multiple associated SNPs reported by Yang et al.
